# Association of the Paediatric Admission Quality of Care score with mortality in Kenyan hospitals: a validation study

**DOI:** 10.1016/S2214-109X(17)30484-9

**Published:** 2018-01-19

**Authors:** Charles Opondo, Elizabeth Allen, Jim Todd, Mike English

**Affiliations:** a Health Services Unit, KEMRI Wellcome Trust Research Programme, Nairobi, Kenya; b Department of Medical Statistics, Faculty of Epidemiology and Population Health, London School of Hygiene & Tropical Medicine, London, UK; c Department of Population Health, Faculty of Epidemiology and Population Health, London School of Hygiene & Tropical Medicine, London, UK; d Department of Epidemiology and Biostatistics, Kilimanjaro Christian Medical University College, Moshi, Tanzania; e Centre for Tropical Medicine and Global Health, Nuffield Department of Medicine, University of Oxford, Oxford, UK

## Abstract

**Background:**

Measuring the quality of hospital admission care is essential to ensure that standards of practice are met and continuously improved to reduce morbidity and mortality associated with the illnesses most responsible for inpatient deaths. The Paediatric Admission Quality of Care (PAQC) score is a tool for measuring adherence to guidelines for children admitted with acute illnesses in a low-income setting. We aimed to explore the external and criterion-related validity of the PAQC score by investigating its association with mortality using data drawn from a diverse sample of Kenyan hospitals.

**Methods:**

We identified children admitted to Kenyan hospitals for treatment of malaria, pneumonia, diarrhoea, or dehydration from datasets from three sources: an observational study, a clinical trial, and a national cross-sectional survey. We extracted variables describing the process of care provided to patients at admission and their eventual outcomes from these data. We applied the PAQC scoring algorithm to the data to obtain a quality-of-care score for each child. We assessed external validity of the PAQC score by its systematic replication in datasets that had not been previously used to investigate properties of the PAQC score. We assessed criterion-related validity by using hierarchical logistic regression to estimate the association between PAQC score and the outcome of mortality, adjusting for other factors thought to be predictive of the outcome or responsible for heterogeneity in quality of care.

**Findings:**

We found 19 065 eligible admissions in the three validation datasets that covered 27 hospitals, of which 12 969 (68%) were complete cases. Greater guideline adherence, corresponding to higher PAQC scores, was associated with a reduction in odds of death across the three datasets, ranging between 9% (odds ratio 0·91, 95% CI 0·84–0·99; p=0·031) and 30% (0·70, 0·63–0·78; p<0·0001) adjusted reduction per unit increase in the PAQC score, with a pooled estimate of 17% (0·83, 0·78–0·89; p<0·0001). These findings were consistent with a multiple imputation analysis that used information from all observations in the combined dataset.

**Interpretation:**

The PAQC score, designed as an index of the technical quality of care for the three commonest causes of admission in children, is also associated with mortality. This finding suggests that it could be a meaningful summary measure of the quality of care for common inpatient conditions and supports a link between process quality and outcome. It might have potential for application in low-income countries with similar disease profiles and in which paediatric practice recommendations are based on WHO guidelines.

**Funding:**

The Wellcome Trust.

## Introduction

The Paediatric Admission Quality of Care (PAQC) score^1^ is a tool for measuring the quality of admission care for treatment of acute illnesses in hospitals in a low-income setting in which in-hospital child mortality is relatively high and quality of hospital care relatively poor. Quality of care is scored against six generic items (panel) encompassing three domains of the process of care—assessment, diagnosis, and treatment—that are clearly articulated in Kenyan guidelines on hospital care for children^2^ derived from those of WHO.^3^ Items are scored 0 or 1 depending on whether or not they have been carried out as recommended. The overall PAQC score is then obtained by summing over the item scores. The maximum (optimal) PAQC score is 6.

Panel **Items included in the PAQC score**

1 Assessment for primary signs and symptoms of illness
2 Assessment for secondary signs and symptoms
3 Additional assessments for complete documentation of illness
4 Diagnosis that includes an illness severity classification consistent with guidelines
5 Treatment suitable for illness severity as recommended in guidelines
6 Correct application of selected treatment as recommended in guidelines

PAQC=Paediatric Admission Quality of Care.

The PAQC score was designed to provide an intuitive, meaningful, and consistent way to quantify the extent to which paediatric admission care meets the standards of technical quality of clinical processes defined in case management guidelines. It currently encompasses malaria, pneumonia, and diarrhoea, which are responsible for about 60% of paediatric admissions in Kenya.^4^ Its intended applications include benchmarking of quality of care, assessment of quality improvement interventions, and steering of improvements in care. To facilitate the score's acceptability to health workers and health policy makers and wider adoption by health service researchers, we now describe the process and results of validation of the PAQC score.

Validation of a measure involves an exploration of its properties to establish whether the decisions and inferences that the measure can lead to are appropriate, meaningful, and useful.^5^ Thus, the validity of a measure can be assessed through its association with the construct it is intended to measure (construct validity), its credibility to its intended audience and users (face validity), how well it correlates with other measures that quantify the same construct (criterion-related validity), and how well it works in real-world situations that are different from those under which it was originally designed or tested (external validity, also referred to as generalisability). Construct, face, and content validity of the PAQC score were addressed during the design stage by using clinical practice guidelines, which define the standards of quality of care, as the basis for the items making up the score.^1^ Generalisability of the PAQC score can be shown through systematic replication of the score in a setting different from that in which it was created.^6^, ^7^

**Research in context**

**Evidence before this study**

Hospital care for children in low-income countries has been poor, and evidence suggests that the use of clinical practice guidelines (CPGs) could help to improve processes of care. In Kenya, CPGs have been developed by adapting WHO guidelines to target initial paediatric care on admission to district hospitals. A package of implementation strategies was shown to be an effective mechanism for promoting adherence to these CPGs in a cluster randomised controlled trial, and CPGs and recommended care practices have been increasingly adopted in routine settings. However, assessment of whether improvements in adherence to guidelines translate to better outcomes has not been possible because approaches to reliably measuring and assessing trends in overall quality of care in this setting are not well developed. We did a literature search on MEDLINE, PubMed, and Embase from inception up to April 13, 2012, using the terms (“design” OR “develop” OR “build” OR “theory” OR “construct” OR “create”) AND (“score” OR “scale” OR “index” OR “measure” OR (“composite” AND “indicator”)) AND “quality of health care” AND (“process” OR “outcome”) AND (“child” OR “infant” OR “newborn”). We aimed to identify studies describing the development of summary measures of quality of care for children in low-income settings, and studies providing descriptions of methodological approaches to developing summary scores for quality-of-care measurement. We found 15 studies describing approaches to the development of summary scores in general, but none applied to quality of care for children in a low-income setting. In earlier work, we therefore set out to develop and describe the Paediatric Admission Quality of Care (PAQC) score, which represents the first step in addressing the problem of a paucity of systematically designed measures of paediatric quality of care in a low-income setting.

**Added value of this study**

This work augments our previous work on the development of the PAQC score. It provides evidence that, in settings with relatively poor adherence to guidelines for conditions that are commonly associated with child mortality in hospital (malaria, dehydration, and pneumonia), a higher PAQC score—indicating improved guideline adherence that is a marker of process quality—is associated with lower inpatient mortality. This finding supports the validity of the PAQC score as a useful summary measure of quality of admission care for three major diseases and suggests that it could be of value to health workers and policy makers seeking an efficient measure of the quality of hospital services for children. This study also provides a framework for the development of similar summary quality measures in other areas of hospital care in which guidelines for standards of care have been established but quality measures are still poorly developed.

**Implications of all the available evidence**

Previous evidence has shown that efforts to implement CPGs could help to improve processes of care. Our findings that increased PAQC scores are associated with reduced risk of inpatient mortality show that such improvements in processes are associated with better outcomes of paediatric admission.

For criterion-related validation, a so-called gold-standard criterion known to correlate with a relevant process or outcome is required. For example, in critical care settings, the acute physiology and chronic health evaluation score (used to classify patients by illness severity in intensive care) and the Glasgow coma scale (used to evaluate level of consciousness in patients with brain trauma) have been validated against mortality risk, which is a relevant criterion in the prognosis of critical care patients.^8^, ^9^, ^10^, ^11^ Similarly, in acute illness, mortality—or, more specifically, survival—is arguably the most relevant criterion to assess whether a score capturing adherence to guidelines has value beyond that of a process measure. The ideal is that the application of specific guideline recommendations avert mortality, at least in low-income settings. The guidelines on paediatric care upon which the PAQC score is based recommend that clinicians providing care to acutely ill children identify signs and symptoms of illness to make a diagnosis and classify illness severity.^2^ This is because signs such as altered level of consciousness, respiratory distress, nutritional status, signs of anaemia, and inability to feed, which the guidelines direct clinicians to look out for, are associated with death in this inpatient population.^12^, ^13^ The guidelines also make recommendations on effective treatments for the most common illnesses—including malaria, pneumonia, and diarrhoea, which are the focus of this study—and how to use these treatments. The PAQC score measures clinicians' fidelity to guideline-recommended processes of care and effective treatment. Therefore, we hypothesised that, in settings with high and potentially avoidable mortality, care corresponding to higher scores would be associated with lower odds of mortality when adjusting for other factors that might be prognostic for death during an acute admission episode.

Thus, the overall aim of this study was to investigate the validity of the PAQC score. Specifically, we aimed to determine whether the score was associated with inpatient mortality to establish its criterion-related validity, linked to the hypothesis that process quality can influence outcomes;^14^ and to explore the external validity (generalisability) of the score through its systematic replication in a set of hospitals widely typical of the Kenyan setting.

## Methods

### Data sources

We included data from three sources in this validation study: a Kenyan district hospitals (KDH) study,^15^ an observational dataset linked to a pneumonia trial,^16^ and a Ministry of Health (MoH) cross-sectional survey of 22 training hospitals in Kenya.^17^ Although some hospitals were included in more than one of the datasets, the data from the different studies were collected during non-overlapping periods, preventing duplication of data. All data come from case records of children admitted to hospital for treatment of acute illness, and describe the care provided by the admitting clinician and subsequent survival outcome. Methods for collecting these data have been described elsewhere.^15^, ^18^ Although the quality of health records in low-income countries is generally reported to be poor, paediatric records in Kenya might be somewhat better because efforts have been made to promote better documentation, with some success.^19^, ^20^ We only included children whose case records indicated a diagnosis of malaria, pneumonia, or diarrhoea in the analysis because the PAQC score has only been developed for these conditions. All conditions were clinically diagnosed, and in the case of malaria, frequently (but not universally) supported by a blood slide done by the hospital's own laboratory. Checking the quality of these tests was not part of the studies contributing the data.

The KDH study^15^ included observations from 12 036 children younger than 5 years admitted to eight hospitals across Kenya for treatment of acute illnesses over a 59-month period between Feb 1, 2005, and Dec 30, 2009. The study investigated the effect of an intervention involving training health workers to provide evidence-based care according to practice guidelines, with supervision and feedback, to improve the quality of inpatient care provided to children. The context, conduct, and findings of this study have been described in detail elsewhere.^15^, ^21^, ^22^ Although data from the KDH study have been used to investigate properties of the PAQC score (including its distribution and responsiveness to intervention),^1^ no previous validation of the score has been made on these data.

The pneumonia trial from which the linked observational dataset was obtained was a randomised, controlled, multicentre, non-inferiority trial^16^ of oral amoxicillin compared with injected penicillin for the treatment of severe pneumonia in children under 5 years old. The data include all children with illnesses not requiring surgical treatment who were admitted to the paediatric wards of seven public hospitals at the same time as those randomised to the trial, and were collected to explore whether those in the trial were systematically different from those receiving routine care.^23^ These data were collected between Sept 12, 2011, and Aug 15, 2013, and include admissions occurring between Jan 1, 2011, and May 25, 2013.

In the MoH cross-sectional survey,^17^ data on process of care were collected from paediatric case records identified from inpatient registers starting from admissions on May 31, 2012, and going back in time until a sample of approximately 60 records per unit per hospital was obtained.

No major changes were made to clinical guideline recommendations over the time period covered by these datasets. The same data collection tool was used in all three studies because Kenya has promoted the use of standard case records,^19^, ^20^ which enabled the use of standard definitions of variables derived from the three datasets.

All three studies from which data were derived received ethical approval from the Kenya Medical Research Institute National Ethical Review and Scientific Review Committees. Ethical approval was granted for confidential abstraction of data from archived case records without individuals' consent. This work used de-identified data for secondary analysis and did not require additional approval.

### Assessment of external validity

We assessed external validity (generalisability) of the PAQC score by its systematic replication in the pneumonia trial dataset and the MoH survey dataset. The PAQC score's six generic process-of-care indicators measure whether guideline-recommended processes of care for the three diseases of interest were undertaken by the clinician attending to the child. It can be calculated at the individual patient level separately for each of the three illnesses or as a combined score for children with multimorbidity.^1^

We identified key variables needed to generate the indicators and calculate the patient-level PAQC score in the KDH dataset—the score development dataset—and matched these to corresponding variables in the validation datasets. Where necessary, we renamed and recoded the matched variables to maintain consistency with those in the development dataset. We then generated the patient-level PAQC score in the validation datasets using the previously described procedure.^1^

### Variables for criterion-related validation

The main exposure variable for criterion-related validation was the patient-level PAQC score. The validation outcome variable was inpatient mortality at any point of the admission episode. Mortality was a preferred outcome for validation because it is an objective and relevant outcome of hospitalisation.

The age and sex of the children included in the data were identified a priori as likely confounding variables. The number of comorbidities (among the three diseases of interest), illness severity classification as defined by the guidelines, and duration of their hospital admission were also expected to be associated with the outcome; for example, the risk of mortality tends to be higher in multimorbidity and more severe illness, whereas shorter duration of hospitalisation might be associated with mortality because most inpatient deaths in this setting tended to occur within the first 48 h of admission.^4^

### Statistical analysis

We fitted hierarchical logistic regression models separately in each of the three datasets to explore the association between mortality and the PAQC score. These models systematically adjusted for the number of comorbidities, illness severity, and the duration of hospital admission, with age and sex retained in the models as a-priori likely confounders. We adjusted for trial arm and survey number in the KDH dataset, which was derived from an intervention study. The HIV status of children was not known and therefore could not be adjusted for. We used likelihood ratio tests to investigate the presence of linear trend in mortality across levels of the score. We estimated hospital-level random effects to adjust for clustering of observations within each hospital. We examined the goodness of fit of the hierarchical models by plotting the predicted hospital-level random effects versus their rank to check that the assumption of normality was not violated. We handled missing data by listwise deletion, resulting in a complete case analysis. We obtained a pooled estimate of the adjusted association of the PAQC score with mortality in all three datasets by individual participant data meta-analysis, using a hierarchical logistic regression with adjustment for clustering within hospital and random slope for study dataset. To assess the robustness of our conclusions to missingness of data, we used multiple imputation with chained equations appropriate for the distributions of the affected variables to create imputed datasets.^24^ We fitted models to the imputed datasets and compared the results with those obtained in the complete case analysis. We determined the required number of imputed datasets using the suggested rule of thumb that it should be at least a hundred times the largest fraction of missing information about coefficient estimates due to non-response.^25^ We did all data management and analysis using Stata version 13.1.

### Data sharing

Data for this study were collected as part of a study approved by the Kenyan Medical Research Institute (KEMRI) Scientific and Ethical Review Committee, and are archived by the KEMRI Wellcome Trust Research Programme (KWTRP). Requests for any data access can be made to the KWTRP Data Governance Committee.

### Role of the funding source

The funders had no role in the design, conduct, analysis, writing, or submission of this manuscript. The corresponding author had full access to the data and took the decision, in conjunction with coauthors, to submit this manuscript for publication.

## Results

**Figure 1:**
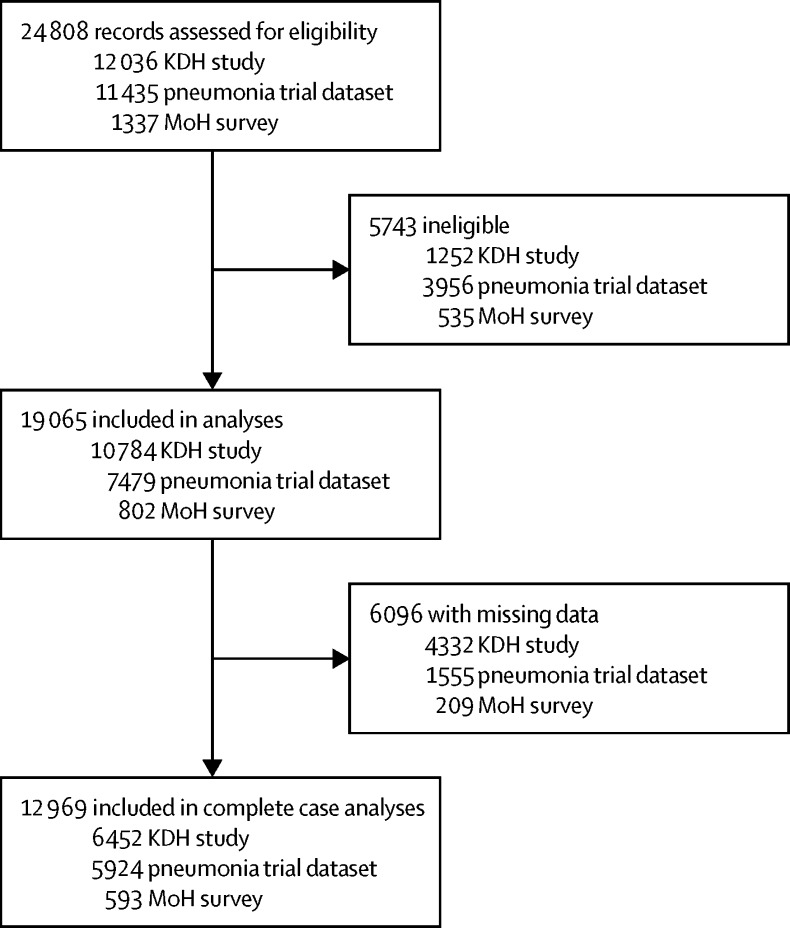
Study profile KDH=Kenyan district hospitals. MoH=Ministry of Health.

We found 24 808 records of children younger than 5 years admitted between Feb 1, 2005, and May 25, 2013, across the 25 hospitals included in the three datasets. Of these, 19 065 (77%) had a diagnosis of malaria, pneumonia, or diarrhoea and were eligible for inclusion in all subsequent analyses, and 12 969 (68%) of the eligible children had complete data on all variables of interest (figure 1).

Most eligible records came from the KDH study (57%), with the pneumonia trial dataset accounting for 39% of case records and the MoH dataset providing the remaining 4% (figure 1). Mortality ranged between 5% and 8% across the datasets, with up to 6% of children having an unknown outcome, which includes those removed from hospital by their parent or guardian against medical advice (table 1); the distribution of mortality by participant characteristics in each dataset is shown in the appendix. Most admission episodes lasted less than a week (table 1). Pneumonia was the single most common diagnosis, responsible for between 49–76% of admissions (table 1). The distribution of the PAQC score in the validation datasets was similar to that of the development dataset; notably, scores of zero were relatively uncommon (table 1). Multimorbidity was relatively rare in the MoH survey data, affecting only 58 (7%) children (table 1). In subsequent regression modelling of the MoH survey data, we excluded children with three diagnoses (<1%) to avoid problems with parameter estimation caused by sparse data.

**Table 1:** Characteristics of outcome and exposure variables across the three datasets

		**District hospitals study (n=10 784)**	**Pneumonia trial dataset (n=7479)**	**Ministry of Health survey (n=802)**
Outcome, n (%)				
	Alive	9697 (90%)	6673 (89%)	740 (92%)
	Dead	821 (8%)	385 (5%)	43 (5%)
	Unknown (missing)	266 (2%)	421 (6%)	19 (2%)
PAQC score, n (%)				
	0	420 (4%)	65 (1%)	9 (1%)
	1	2467 (23%)	819 (11%)	54 (7%)
	2	1919 (18%)	1514 (20%)	156 (19%)
	3	1530 (14%)	1510 (20%)	140 (17%)
	4	2197 (20%)	2052 (27%)	201 (25%)
	5	1505 (14%)	1215 (16%)	152 (19%)
	6	746 (7%)	304 (4%)	90 (11%)
PAQC score, mean (SD)		2·93 (1·7)	3·27 (1·4)	3·60 (1·5)
Age in years, mean (SD)		1·40 (1·1)	1·88 (2·1)	1·73 (1·6)
Sex, n (%)				
	Male	5121 (47%)	4087 (55%)	461 (57%)
	Female	4227 (39%)	3223 (43%)	329 (41%)
	Not recorded (missing)	1436 (13%)	169 (2%)	12 (1%)
Diagnosis, n (%)				
	Diarrhoea or dehydration	732 (7%)	1381 (18%)	34 (4%)
	Malaria	4821 (45%)	1236 (17%)	158 (20%)
	Pneumonia	5231 (49%)	4862 (65%)	610 (76%)
Number of diseases diagnosed, n (%)				
	Any one	6150 (57%)	6392 (85%)	744 (93%)
	Any two	4188 (39%)	1063 (14%)	56 (7%)
	All three	446 (4%)	24 (<1%)	2 (<1%)
Severity, n (%)				
	Lowest	2446 (23%)	1121 (15%)	65 (8%)
	Intermediate	2637 (24%)	3377 (45%)	347 (43%)
	Highest	2796 (26%)	2312 (31%)	249 (31%)
	Unknown (missing)	2905 (27%)	669 (9%)	141 (18%)
Duration of admission in days, median (IQR)		3 (2–5)	3 (2–5)	4 (2–6)
Observations per hospital, median (range)		1395 (666–1869)	1206 (259–1612)	36 (16–55)
Group, n (%)				
	Control	3802 (35%)	··	··
	Intervention	6982 (65%)	··	··
Survey, n (%)				
	Baseline	2188 (20%)	··	··
	First follow-up	1886 (17%)	··	··
	Second follow-up	1922 (18%)	··	··
	Endpoint	2480 (23%)	··	··
	First post-intervention	1084 (10%)	··	··
	Second post-intervention	1223 (11%)	··	··
	Unknown (missing)	1 (<1%)	··	··

PAQC=Paediatric Admission Quality of Care.

**Figure 2:**
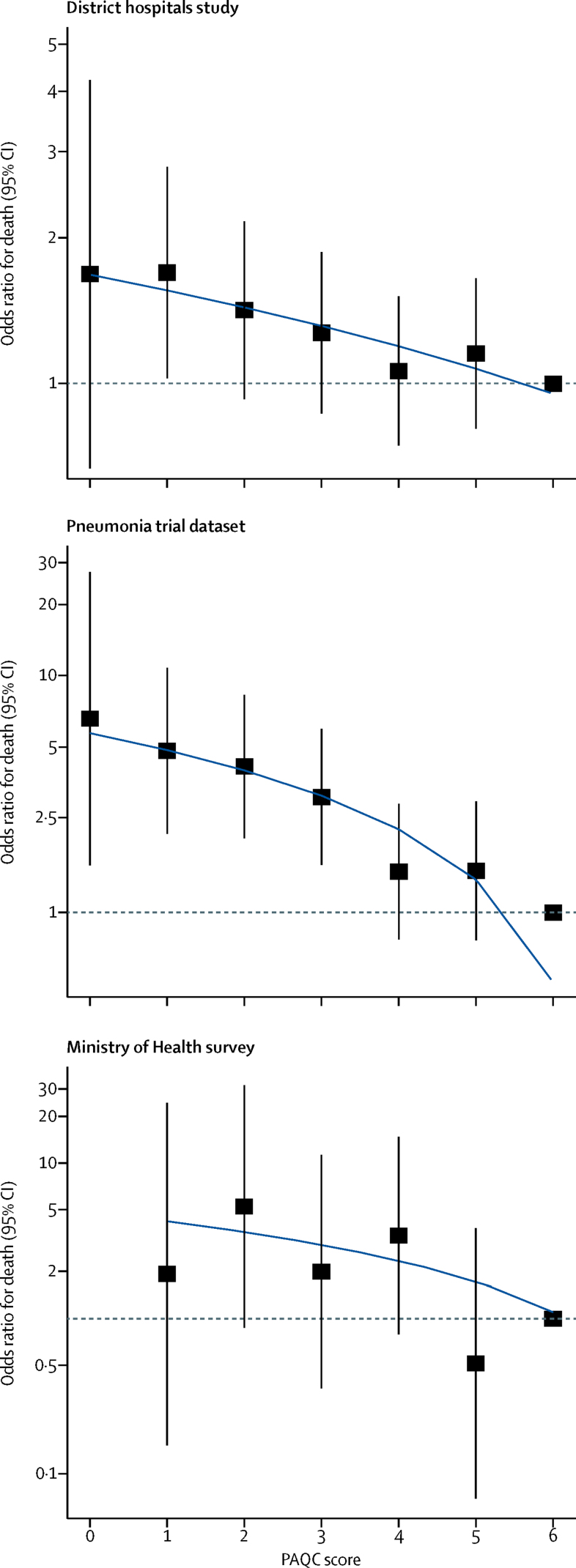
Odds ratios for mortality across levels of the PAQC score Solid black squares are odds ratios with 95% CIs (black vertical lines) corresponding to PAQC scores, with a score of 6 as the reference category. Blue lines show linear trends in odds ratios, obtained from predictions of a linear regression; these lines can curve as the odds ratio tends towards 1 because the y-axis is on a log scale. Dotted grey horizontal lines indicates odds ratio of 1 on the y-axis. PACQ=Paediatric Admission Quality of Care.

We observed in all three datasets a linear trend of declining odds of mortality with increasing PAQC scores (figure 2). For the individual datasets, hierarchical logistic regression of complete cases allowing for clustering at hospital level showed strong evidence of a reduction in the odds of mortality in children whose care corresponded to higher PAQC scores, after adjusting for age, sex, illness severity, duration of hospital admission, multimorbidity, and, in the district hospitals study data, survey and randomised group allocation (table 2). We found no evidence of collinearity between any of the covariates. Likelihood ratio tests suggested a better model when fitting the score as a continuous rather than categorical variable (results not shown). There was evidence of a reduction in adjusted odds of death per unit increase in PAQC score (table 2). We found evidence of heterogeneity of odds ratios across studies (*I*^2^=86·2%, p=0·0007). The pooled adjusted estimate across all datasets, based on the individual participant data meta-analysis, was a 17% reduction in odds of death per unit increase in the PAQC score (table 2). We found no evidence of departure from linearity in the association between PAQC score and mortality (quadratic term odds ratio 1·00, 95% CI 0·98–1·04; p=0·745).

**Table 2:** Estimates of the association of the PAQC score with mortality

	**Crude estimates**			**Adjusted estimates**		
	Number of participants	Odds ratio (95% CI)	p value	Number of participants	Odds ratio (95% CI)	p value
District hospitals study*	10 517	0·97 (0·92–1·02)	0·267	6452	0·91 (0·84–0·99)	0·031
Pneumonia trial dataset	7058	0·86 (0·79–0·92)	0·0001	5924	0·70 (0·63–0·78)	<0·0001
Ministry of Health survey	775	0·91 (0·71–1·15)	0·422	587	0·71 (0·52–0·98)	0·038
Pooled estimates†	18 359	0·98 (0·94–1·02)	0·232	12 969	0·83 (0·78–0·89)	<0·0001

Odds ratios are per unit increase in PAQC score. PAQC=Paediatric Admission Quality of Care.

* Estimates from the model of the district hospitals study data are partially adjusted for survey and randomised group allocation to make them more comparable to the other studies, which were not conducted across multiple surveys or did not entail randomised group allocations.

† Pooled estimate from individual participant data meta-analysis adjusted for child's age, sex, illness severity, duration of admission, and multimorbidity.

In the analysis using the imputed datasets, the magnitudes of the adjusted associations of the score with mortality were slightly reduced; however, the direction and strength of evidence of association were similar to those from the complete case analysis (appendix). The non-missing characteristics of the observations whose missing data were imputed (ie, the observations removed from the complete case analysis by listwise deletion) were similar to the overall characteristics across the datasets; however, these observations had relatively lower PAQC scores (appendix).

## Discussion

This study explored the external validity and criterion-related validity of the PAQC score, which measures whether the care given to children admitted to hospital with acute illnesses complies with guidelines. We sought first to systematically replicate the score in two new datasets to investigate its external validity. We have shown that the PAQC score can be applied to observational datasets in which efforts have been made to promote standard definitions of clinical variables. When applied to such datasets, the PAQC score suggests considerable variability in process quality and might then help to direct improvement efforts and contrast performance across time and place.

We subsequently investigated the PAQC score's association with mortality using regression analysis to test the hypothesis that, as a measure of the process of care based on evidence-informed guidelines, the PAQC score should be associated with outcomes. With the exception of the PAQC score, all variables in the observations with missing data had similar distributions to those from the complete observations in all datasets. This result was expected, because poor documentation of care—a potential sign of poor quality of care—would correspond to lower scores. Nevertheless, results of the multiple imputation were consistent with the complete case analysis.

We observed a linear reduction in adjusted odds of death with increasing scores, consistent with our hypothesis about the PAQC score's criterion-related validity, which is linked to the concept that improved process should be associated with improved outcomes. The association between the score and mortality was consistent, but of varied magnitude, across the three datasets, providing evidence of the external validity of the score. The magnitudes of the association varied across the datasets, ranging from 9% to 30% adjusted reduction in odds of death for every unit increase in the PAQC score, with an overall pooled estimate of 17% reduction. These differences might be indicative of context-specific variation in processes and outcomes, including differences in hospital size, admission numbers, staffing, level in the referral chain, presence of other interventions, and even baseline mortality rates and level of quality of care. In any case, the PAQC score is not intended to predict outcomes, and as quality of care improves we would expect to see no association with mortality. As such, the estimates of the magnitude of the association should be interpreted cautiously.

The characteristics of the PAQC score shown in this study and elsewhere^1^ suggest that it could be an attractive tool in several situations in which quality of care is still relatively low and quality measurement is still poorly developed. For example, in routine quality assessment and reporting in Kenya and many other low-income countries, measurement has so far focused on availability of resources because shortfalls in these areas have prevented meaningful progress in improving outcomes.^26^ With the development in the past 10 years of clinical practice guidelines^27^, ^28^ and their increasing adoption, intuitive, relevant, and validated measures are needed to monitor whether clinicians are providing recommended forms of care and to identify aspects of care that might need improvement. Scores based on guideline adherence might be particularly relevant in places where most care on admission is given by junior medical staff with very little paediatric training and experience. Another potential use of the PAQC score is as a process outcome in trials and studies seeking to measure quality of care as an outcome. Such studies have previously relied on either complex ad-hoc measures—such as the index implemented in the multicountry evaluation of the integrated management of childhood illnesses^29^—or indicator sets that are varied across illnesses^30^ and studies, thereby making effective comparisons of findings difficult.^31^ However, use of the PAQC score will be greatly facilitated by the adoption of common clinical terms and ideally standardised medical records, possibly as part of electronic medical record systems.

A wide variability in both quality of care provided and mortality across hospitals has been observed in these data and elsewhere.^4^ When quality of care is poor and adverse outcomes relatively common, fairly small improvements in care can lead to a reduction in the prevalence of adverse outcomes. However, over time, as care improves everywhere, further marginal increases in quality might result in only minor additional improvements in outcomes, which could make associations between measures of the process of care and outcomes more difficult to detect. For example, if admission care adhered to guideline recommendations in almost all cases, corresponding to very high mean PAQC scores, mortality will still occur.

Further research could therefore explore the formative validity of the PAQC score—that is, its ability to steer progress towards maintaining high standards of care provided by junior staff admitting patients while the challenges of delivering sustained quality care over the entire admission period are tackled.

Our study had several limitations. As with all observational studies, the potential for confounding due to unmeasured factors (for example, comorbidities such as HIV status that are not included in the clinical record or in this analysis) and unobserved outcomes (such as deaths after discharge) remains. The PAQC score is based entirely on admission documentation and treatment in the first 48 h, leaving room for later events to influence outcomes.

Possible sources of bias include the possibility of situations in which assessments were incomplete or not recorded because clinicians directed their efforts to the resuscitation of a child who was severely ill at presentation. Missing data was another possible source of bias. A complete case analysis can give biased estimates if the assumption of missingness completely at random is violated. However, multiple imputation analysis, which assumed missingness at random—a more relaxed assumption than missingness completely at random—led to similar conclusions as the complete case analysis.

All data came from one country, which has fairly widespread agreement on clinical terminology and in which good dissemination of national guidelines has occurred, and therefore might limit the generalisability of our findings. Additionally, our analysis has focused on only three illnesses. However, the generic structure of the PAQC score suggests that it could be applicable to most other acute childhood illnesses—and indeed other inpatient settings—in which higher scores corresponding to better care according to guidelines are expected to correspond with reduced odds of adverse outcomes.

Although our study has shown that the score follows key principles that have been proposed for the development and application of quality-of-care measures,^32^ we have not evaluated its cost, both in terms of time and resource allocation, against any potential benefit to receivers, providers, and planners of health care. Therefore, some scope exists for further research to address these issues.

In conclusion, our study provides evidence that the PAQC score can be applied to quality-of-care measurement in a variety of paediatric inpatient settings, and that it is consistently associated with an objective outcome of care across these settings. To the best of our knowledge, our analysis is the first to show the validity of a composite quality-of-care measure in a low-income setting using mortality as the criterion for validation, based on the idea that process quality should be related to outcomes. Our findings strengthen the face validity of the PAQC score, and will hopefully reassure policy makers and practitioners of its usefulness as a meaningful measure of process quality that could be used to contrast performance across place and time.
