# Supplementary material for: Association of the Paediatric Admission Quality of Care score with mortality in Kenyan hospitals: a validation study
Source: Lancet Glob Health. 2018 Jan 19;6(2):e203–10. doi: 10.1016/S2214-109X(17)30484-9 (PMC5785367; doi:10.1016/S2214-109X(17)30484-9)
Supplement: Supplementary appendix [file mmc1.pdf]

THE LANCET
Global Health

Supplementary appendix

This appendix formed part of the original submission and has been peer reviewed. We post it as supplied by the authors.

Supplement to: Opondo C, Allen E, Todd J, English M. Association of the Paediatric Admission Quality of Care score with mortality in Kenyan hospitals: a validation study. *Lancet Glob Health* 2018; **6:** e203–10.

Appendix Table 1: **Distribution of the outcome across levels of categorical exposure variables**

	**District hospitals study (n=10,784)**		**Pneumonia trial linked observation (n=7,479)**		**Ministry of Health survey (n=802)**	
	Category, n (% of total)	Died, n (% of category)	Category, n (% of total)	Died, n (% of category)	Category, n (% of total)	Died, n (% of category)
PAQC score						
0	420 (3·9%)	33 (7·9%)	65 (0·9%)	4 (6·2%)	9 (1·1%)	0 (0·0%)
1	2467 (22·9%)	166 (6·7%)	819 (11·0%)	41 (5·0%)	54 (6·7%)	1 (1·9%)
2	1919 (17·8%)	142 (7·4%)	1514 (20·2%)	82 (5·4%)	156 (19·5%)	10 (6·4%)
3	1530 (14·2%)	138 (9·0%)	1510 (20·2%)	96 (6·4%)	140 (17·5%)	7 (5·0%)
4	2197 (20·4%)	160 (7·3%)	2052 (27·4%)	86 (4·2%)	201 (25·1%)	18 (9·0%)
5	1505 (14·0%)	123 (8·2%)	1215 (16·3%)	65 (5·4%)	152 (19·0%)	4 (2·6%)
6	746 (6·9%)	59 (7·9%)	304 (4·1%)	11 (3·6%)	90 (11·2%)	3 (3·3%)
Age (years completed)						
0	4927 (45·7%)	503 (10·2%)	3275 (43·8%)	237 (7·2%)	338 (42·1%)	25 (7·4%)
1	2919 (27·1%)	171 (5·9%)	1858 (24·8%)	71 (3·8%)	179 (22·3%)	9 (5·0%)
2	1508 (14·0%)	74 (4·9%)	815 (10·9%)	22 (2·7%)	135 (16·8%)	4 (3·0%)
3	879 (8·2%)	41 (4·7%)	516 (6·9%)	13 (2·5%)	56 (7·0%)	3 (5·4%)
4	551 (5·1%)	32 (5·8%)	381 (5·1%)	10 (2·6%)	64 (8·0%)	2 (3·1%)
5 or more	0 (0·0%)	0 (0·0%)	634 (8·5%)	32 (5·1%)	30 (3·7%)	0 (0·0%)
Sex,						
Male	5121 (47·5%)	370 (7·2%)	4087 (54·7%)	199 (4·9%)	461 (57·5%)	24 (5·2%)
Female	4227 (39·2%)	364 (8·6%)	3223 (43·1%)	178 (5·5%)	329 (41·0%)	19 (5·8%)
Not recorded (missing)	1436 (13·3%)	87 (6·1%)	169 (2·3%)	8 (4·7%)	12 (1·5%)	0 (0·0%)
Diagnosis,						
Diarrhoea/dehydration	732 (6·8%)	69 (9·4%)	1381 (18·5%)	52 (3·8%)	34 (4·2%)	4 (11·8%)
Malaria	4821 (44·7%)	284 (5·9%)	1236 (16·5%)	42 (3·4%)	158 (19·7%)	10 (6·3%)
Pneumonia	5231 (48·5%)	468 (9·0%)	4862 (65·0%)	291 (6·0%)	610 (76·1%)	29 (4·8%)
Number of diseases diagnosed,						
Any one	6150 (57·0%)	462 (7·5%)	6392 (85·5%)	309 (4·8%)	744 (92·8%)	43 (5·8%)
Any two	4188 (38·8%)	311 (7·4%)	1063 (14·2%)	73 (6·9%)	56 (7·0%)	0 (0·0%)
All three	446 (4·1%)	48 (10·8%)	24 (0·3%)	3 (12·5%)	2 (0·3%)	0 (0·0%)
Severity,						
Lowest	2446 (22·7%)	140 (5·7%)	1121 (15·0%)	38 (3·4%)	65 (8·1%)	2 (3·1%)
Intermediate	2637 (24·5%)	228 (8·7%)	3377 (45·2%)	142 (4·2%)	347 (43·3%)	14 (4·0%)

Highest	2796 (25·9%)	273 (9·8%)	2312 (30·9%)	186 (8·0%)	249 (31·1%)	22 (8·8%)
Unknown (missing)	2905 (26·9%)	180 (6·2%)	669 (9·0%)	19 (2·8%)	141 (17·6%)	5 (3·6%)
Duration of admission						
Less than 1 day	404 (3·8%)	193 (47·8%)	314 (4·2%)	75 (23·9%)	19 (2·4%)	6 (31·6%)
1 day	1208 (11·2%)	183 (15·2%)	1153 (15·4%)	110 (9·5%)	75 (9·4%)	9 (12·0%)
2 days	1978 (18·3%)	88 (4·5%)	1391 (18·6%)	37 (2·7%)	156 (19·5%)	6 (3·9%)
3 days	1561 (14·5%)	48 (3·1%)	1058 (14·2%)	34 (3·2%)	115 (14·3%)	5 (4·4%)
4 days	1282 (11·9%)	42 (3·3%)	764 (10·2%)	18 (2·4%)	116 (14·5%)	4 (3·5%)
5 days or more	3168 (29·4%)	192 (6·1%)	2251 (30·1%)	77 (3·4%)	256 (31·9%)	7 (2·7%)
Unknown (missing)	1183 (11·0%)	75 (6·3%)	548 (7·3%)	34 (6·2%)	65 (8·1%)	6 (9·2%)
Group			N/A		N/A	
Control	3802 (35·3%)	239 (6·3%)				
Intervention	6982 (64·7%)	582 (8·3%)				
Survey			N/A		N/A	
Baseline	2188 (20·3%)	124 (5·7%)				
1st follow-up	1886 (17·5%)	131 (7·0%)				
2nd follow-up	1922 (17·8%)	154 (8·0%)				
End-point	2480 (23·0%)	198 (8·0%)				
1st post-intervention	1084 (10·1%)	111 (10·2%)				
2nd post-intervention	1223 (11·3%)	103 (8·4%)				
Unknown (missing)	1 (0·0%)	0 (0·0%)				

Appendix Table 2: **Comparing adjusted* estimates of the association of the PAQC score with mortality derived from complete case analysis *vs* multiple imputation for missing data**

	**Complete case analysis**				**Multiple imputation**			
**Dataset**	**n**	**OR**	**95% CI**	**p value**	**n**	**OR**	**95% CI**	**p value**
District hospitals study	6452	0·91	0·84–0·99	0·031	10 783	0·92	0·86–0·98	0·010
Pneumonia trial observation	5924	0·70	0·63–0·78	<0·0001	7479	0·76	0·69–0·83	<0·0001
Ministry of Health survey	587	0·71	0·52–0·98	0·038	793	0·75	0·56–0·99	0·041
**Pooled estimates†**	**12 969**	**0·83**	**0·78–0·89**	**<0·0001**	**19 065**	**0·91**	**0·87–0·95**	**<0·0001**

*All models are adjusted for adjusted for child's age, sex, illness severity, duration of admission and multimorbidity. Additionally, estimates from the model of the district hospitals study data are further adjusted for survey and randomised group allocation. †Pooled estimate from IPD meta-analysis.

Appendix Table 3: **Characteristics of outcome and exposure variables across the three datasets in observations removed from the complete case analysis by listwise deletion**

	**District hospitals study (n=4332)**		**Pneumonia trial linked observation (n=1555)**		**Ministry of Health survey (n=215)**	
Outcome, n (%)						
Alive	3 786	(87·4%)	1073	(69·0%)	185	(86·1%)
Dead	280	(6·5%)	61	(3·9%)	11	(5·1%)
Unknown (missing)	266	(6·1%)	421	(27·1%)	19	(8·8%)
PAQC score, n (%)						
0	326	(7·5%)	25	(1·6%)	9	(4·2%)
1	1852	(42·8%)	303	(19·5%)	31	(14·4%)
2	1033	(23·9%)	573	(36·9%)	97	(45·2%)
3	413	(9·5%)	239	(15·4%)	37	(17·2%)
4	362	(8·4%)	244	(15·7%)	26	(12·1%)
5	239	(5·5%)	135	(8·7%)	13	(6·1%)
6	107	(2·5%)	36	(2·3%)	2	(0·9%)
PAQC score, mean (SD)	1·95	(1·4%)	2·59	(1·4%)	2·40	(1·23%)
Age in years, mean (SD)	1·41	(1·1%)	1·89	(2·3%)	1·50	(1·4%)
Sex, n (%)						
Male	1 583	(36·5%)	764	(49·1%)	130	(60·5%)
Female	1 313	(30·3%)	622	(40·0%)	73	(34·0%)
Not recorded (missing)	1 436	(33·2%)	169	(10·9%)	12	(5·6%)
Diagnosis, n (%)						
Diarrhoea/dehydration	143	(3·3%)	197	(12·7%)	4	(1·9%)
Malaria	2 342	(54·1%)	139	(8·9%)	16	(7·4%)
Pneumonia	1847	(42·6%)	1219	(78·4%)	195	(90·8%)
Number of diseases diagnosed, n (%)						
Any one	2827	(65·3%)	1408	(90·6%)	205	(95·4%)
Any two	1406	(32·5%)	141	(9·1%)	9	(4·2%)
All three	99	(2·3%)	6	(0·4%)	1	(0·5%)
Severity, n (%)						
Lowest	518	(12·0%)	157	(10·1%)	15	(7·0%)
Intermediate	477	(11·0%)	455	(29·3%)	38	(17·7%)

Highest	432	(10·0%)	274	(17·6%)	21	(9·8%)
Unknown (missing)	2 905	(67·1%)	669	(43·0%)	141	(65·6%)
Duration of admission in days, median (IQR)	3	(2–5)	3	(2–5)	4	(2–6)
Observations per hospital, median (range)	534	(350–04)	184	(69–423)	8	(1–28)
Group, n (%)			N/A		N/A	
Control	1993	(46·0%)				
Intervention	2339	(54·0%)				
Survey, n (%)			N/A		N/A	
Baseline	1861	(43·0%)				
1st follow-up	669	(15·4%)				
2nd follow-up	513	(11·8%)				
End-point	689	(15·9%)				
1st post-intervention	146	(3·4%)				
2nd post-intervention	453	(10·5%)				
Unknown (missing)	1	(0·02%)				
